# Inhibitor of apoptosis proteins are required for effective fusion of autophagosomes with lysosomes

**DOI:** 10.1038/s41419-018-0508-y

**Published:** 2018-05-09

**Authors:** Sylwia Gradzka, Oliver S. Thomas, Oliver Kretz, Aladin Haimovici, Lazaros. Vasilikos, Wendy Wei-Lynn Wong, Georg Häcker, Ian E. Gentle

**Affiliations:** 1 Institute of Medical Microbiology and Hygiene, University Medical Center Freiburg, Freiburg, Germany; 2 Faculty of Medicine, University of Freiburg, Freiburg, Germany; 3 Renal Division, University Medical Center Freiburg, Freiburg, Germany; 4 Department of Neuroanatomy, University Freiburg, Freiburg, Germany; 5 Department of Medicine, University Medical Center Hamburg-Eppendorf, Hamburg, Germany; 6 Institute of Experimental Immunology, University of Zurich, Zurich, Switzerland

## Abstract

Inhibitor of Apoptosis Proteins act as E3 ubiquitin ligases to regulate NF-κB signalling from multiple pattern recognition receptors including NOD2, as well as TNF Receptor Superfamily members. Loss of XIAP in humans causes X-linked Lymphoproliferative disease type 2 (XLP-2) and is often associated with Crohn’s disease. Crohn’s disease is also caused by mutations in the gene encoding NOD2 but the mechanisms behind Crohn’s disease development in XIAP and NOD2 deficient-patients are still unknown. Numerous other mutations causing Crohn’s Disease occur in genes controlling various aspects of autophagy, suggesting a strong involvement of autophagy in preventing Crohn’s disease. Here we show that the IAP proteins cIAP2 and XIAP are required for efficient fusion of lysosomes with autophagosomes. IAP inhibition or loss of both cIAP2 and XIAP resulted in a strong blockage in autophagic flux and mitophagy, suggesting that XIAP deficiency may also drive Crohn’s Disease due to defects in autophagy.

## Introduction

IAPs are ubiquitin ligases that regulate the activity of TNF Super Family Receptors (TNFSFR), TLRs and NOD receptors. By attaching ubiquitin onto substrates such as RIPK1 they regulate the activation of NF-κB and determine the outcome of signals from these receptors. Their inhibition results in skewing of signals towards death and also production of an inflammatory cytokine response^1–4^. The three best characterised and functionally related IAP members are cIAP1, cIAP2 and XIAP. cIAP1 and cIAP2 act together in complex with TRAF2 and TRAF3^5–7^. Loss of cIAP1 leads to defective NF-κB signals from partner receptors such as TNFR1 and additional activation of non-canonical NF-κB^1^. Less is known about XIAP and its regulation other than it is required for NF-κB signals from NOD2 receptor due to its ubiquitylation activity towards RIPK2^8^.

Genetic loss of cIAP1, cIAP2 and XIAP results in severe systemic inflammation characterised by massive increases in many cytokines including TNF and IL-1β ^2^. IAP antagonist drugs are also able to trigger activation of NLRP3 inflammasomes in LPS primed macrophages^3^. In both cases there appears to be an important role for XIAP, in addition to cIAP1 and cIAP2, in suppressing this inflammatory cascade suggesting some redundancy in the function of IAPs.

Humans with mutations in XIAP often develop Crohn’s disease (CD) but may also suffer from X-linked lymphoproliferative disease 2 (XLP-2)^9–12^. NOD2 is also commonly mutated in CD patients^13–16^. Although mutations in XIAP affect the activation of NOD2, the molecular mechanisms behind CD in these mutations are still not clear. Another gene commonly mutated in CD is the autophagy gene ATG16L1^17^. NOD2 and Atg16L1 are also functionally linked with NOD2 being required for recruiting ATG16L1 to internalised bacteria such as *Salmonella* during xenophagy, the targeted autophagy of invading bacteria^18^. CD associated mutations in NOD2 and Atg16L1 were also shown to block autophagy induction by NOD2 and reduce the xenophagy of invading *Salmonella*^19^. Defects in Atg16L1 or NOD2 also increased the replication of adherent-invasive *Escherichia coli* (AIEC) in macrophages, resulting in enhanced cytokine production. Conversely, induction of autophagy in NOD2^−/−^ macrophages reduced survival of AIEC and cytokine production^20^. Together these data suggest that autophagy is a key pathway linking NOD2 and ATG16L1 in the development of Crohn’s disease and hint that XIAP may also have some role in autophagy regulation.

Autophagy is a highly conserved pathway for recycling cellular components in times of nutrient limitation. Autophagosomes are formed around cellular components such as bulk cytoplasm (macroautophagy), but also specific targets including mitochondria (mitophagy), invasive bacteria (xenophagy) and aggregated proteins (aggrephagy). In each case phagophores, fuse to make autophagosomes that fuse with lysosomes, releasing their contents to be degraded and recycled (reviewed in refs.^21–23^). Autophagy is also linked to many other functions in animals, including regulation of the immune system at various levels such as xenophagy, MHCII presentation, limiting cytokine production in response to infection, and in protein folding diseases such as Alzheimer’s disease (AD) (reviewed in refs.^24–27^).

We here show that both cIAP2 and XIAP promote autophagosome–lysosome fusion and that their loss or inhibition results in accumulation of autophagosomes and lysosomes and defects in mitophagy and xenophagy.

## Results

### IAP antagonism causes accumulation of autophagosomes

To determine if there was any effect of IAP antagonists on autophagy, Mouse Embryonic Fibroblasts (MEFs) were infected with pBabe-mCherry-EGFP-LC3b. GFP fluorescence is sensitive to pH and decreases in the acidic environment of autolysosomes while mCherry retains its fluorescence. Due to this property of GFP, this reporter can be used to assess the rate at which autophagosomes are synthesised and degraded by lysosomes.

**Fig. 1:**
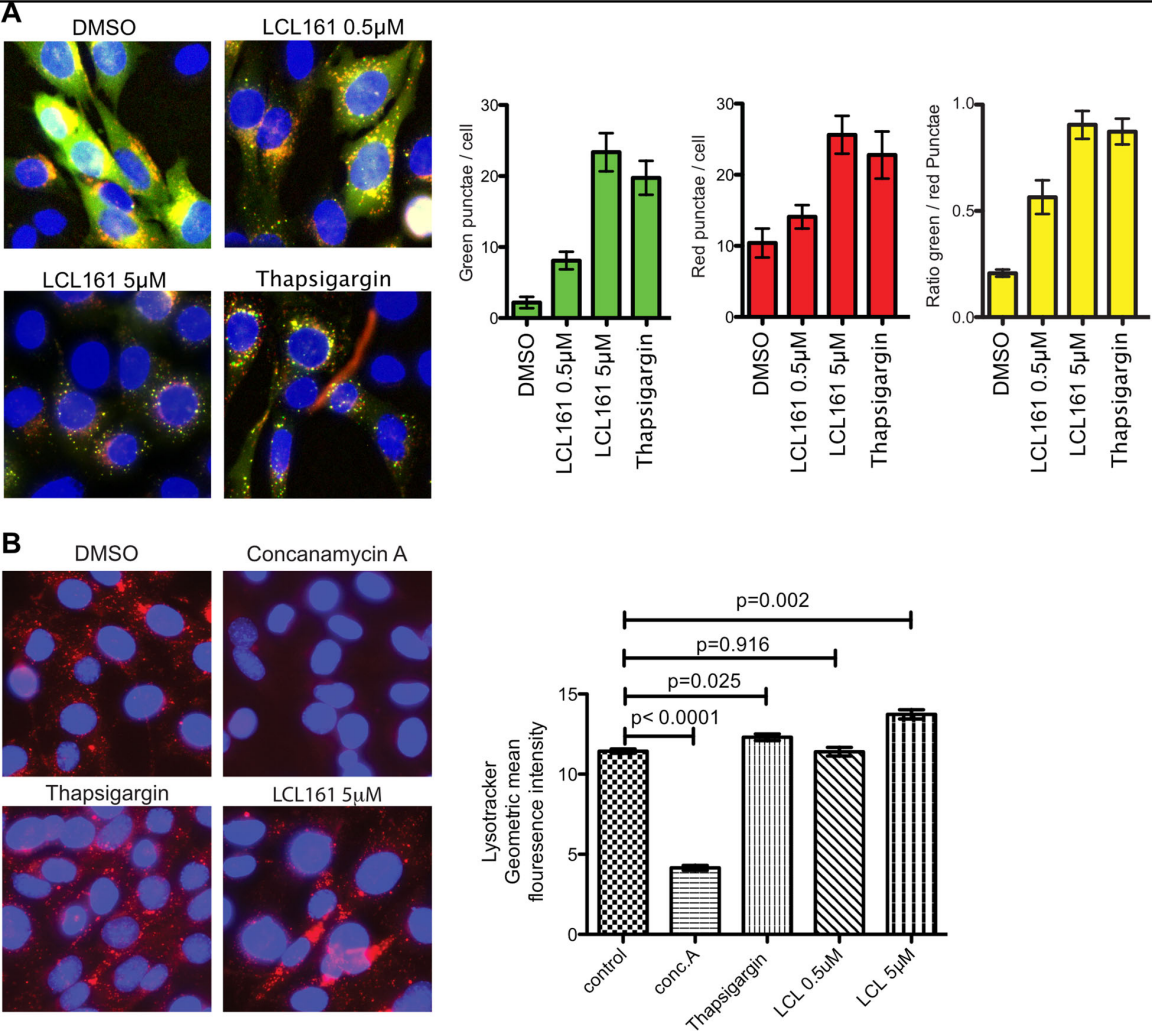
IAP inhibition triggers autophagosome accumulation. **a** Wild type MEFs expressing mcherry-GFP-LC3b were treated with the indicated doses of LCL161or thapsigargin for 6 h. DNA was stained using Hoechst and live Cells were imaged to visualise mCherry, GFP and Hoechst. The number of GFP + puncta and mCherry + puncta were then counted per cell and the ratio of GFP + /mCherry + puncta calculated. Graphs represent the mean and error bars show SEM of at least 3 experiments. **b** IAP antagonism does not block lysosomal acidification. Wild type MEFs were treated with either LCL161 (5 μM), thapsigargin (3 μM) or concanamycin A (2 nM) for 6 h. Cells were stained with lysotracker red and visualised by microscopy or lysotracker intensity measured via flow cytometry. Graphs represent the mean of the geometric mean fluorescence intensity measured by flow cytometry and error bars show SEM of at least 3 independent experiments. P values were calculated using *T*-test in Prism software

Since there is continuous fusion with lysosomes at steady state, there are more single mCherry + puncta in cells expressing GFP-mCherry-LC3b than GFP + mCherry + puncta, and the ratio of GFP + and mCherry + puncta can be used to illustrate this spontaneous fusion. The Cells were treated with the IAP antagonist LCL-161^28^. Upon treatment of MEFs with LCL161, there was a clear accumulation of GFP + puncta at doses as low as 500 nM (Fig. 1a). A slight accumulation of mCherry + puncta could also be detected at this dose but the ratio of GFP/mCherry increased substantially. Treatment with 5 μM LCL161 resulted in more mCherry + GFP + puncta and a ratio of GFP + /mCherry + close to one (Fig. 1a). Similar results were seen with an unrelated IAP antagonist, birinapant that also lead to significant blockage of fusion events, but only at doses of 50 µM or above (Supplementary Fig. 1). Thapsigargin, an irreversible inhibitor of the SERCA calcium pumps of the ER, has been shown to specifically inhibit the fusion of autophagic vesicles with lysosomes, resulting in build up of GFP + mCherry + puncta^29^. As a positive control, MEFs were also treated with thapsigargin, which triggered a build up of GFP + mCherry + puncta as expected. The ratio of GFP + /mCherry + puncta was close to one, reflecting its function in blocking fusion of autophagosomes with lysosomes and similar to the results seen for treatment with IAP antagonists (Fig. 1a). IAP-antagonism therefore causes a build-up of GFP + mCherry + LC3 positive vesicles in MEFs.

This build-up of GFP + mCherry + LC3 positive vesicles could be a result of either a deficiency in turnover of autophagosomes or an increase in activation of autophagy. Defects in autophagosome turnover occur by a number of mechanisms. One possibility is a failure to acidify the autolysosome, resulting in defective lysosomal enzyme function. Such an effect is seen upon the addition of drugs like concanamycin A, an inhibitor of this later stage in autophagy that blocks the activity of ATPase proton pumps in the lysosome, preventing acidification and blocking degradation of lysosomal cargo.

To rule out an effect of IAP antagonists on lysosomal acidification, MEFs were treated with IAP antagonists or concanamycin A to block acidification, or thapsigargin to block autophagosomal fusion with lysosomes. MEFs were then stained with Lysotracker Red, which only fluoresces when lysosomes are acidified. Cells were analysed by microscopy and flow cytometry (Fig. 1b). IAP antagonist treatment did not reduce lysotracker fluorescence whereas Concanamycin A did (Fig. 1b). Both thapsigargin treatment and higher doses of LCL161 enhanced lysotracker staining (Fig. 1b), suggesting a possible accumulation of lysosomes in addition to autophagosomes. IAP antagonists do not therefore affect the acidification of lysosomes, but do lead to a build-up of mCherry + /GFP + positive puncta in a fashion similar to thapsigargin.

**Fig. 2:**
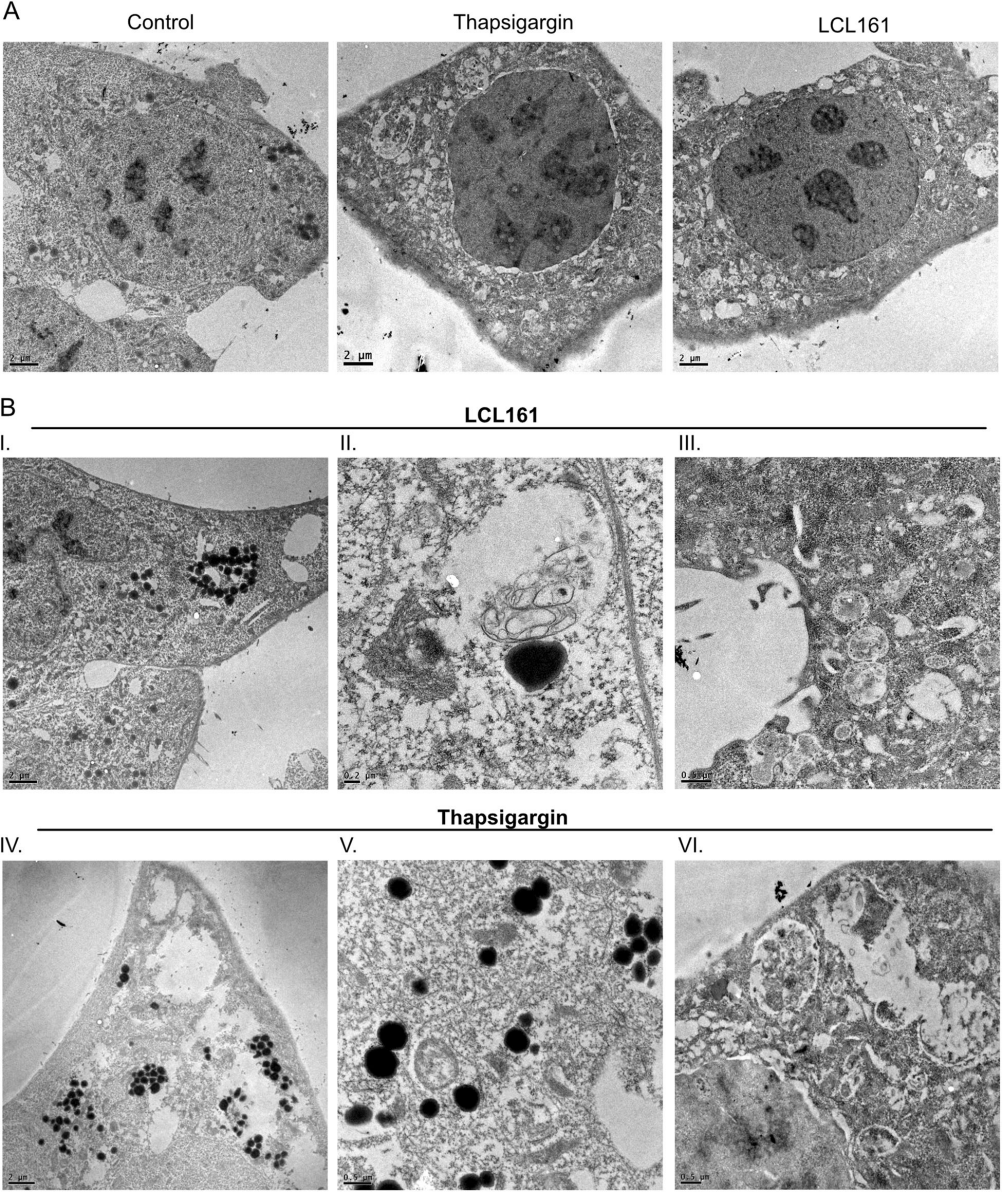
Electron microscopy of IAP antagonist treated cells shows autophagosome and lysosome accumulation. MEFs were treated with thapsigargin (3 μM) or LCL161 (0.5 μM) for 6 h. Cells were fixed and analysed by EM. **a** Overview of whole cells showing increased vesicularization in LCL161 and thapsigargin treated cells. **b** Magnification of LCL161 (I–II) or thapsigargin (IV–VI) treated cells showing I+IV. Accumulation of lysosomes, II+V. Lysosome associated but not fused to autophagosome, III+VI. Autophagosomes with cellular debris inside

**Fig. 3:**
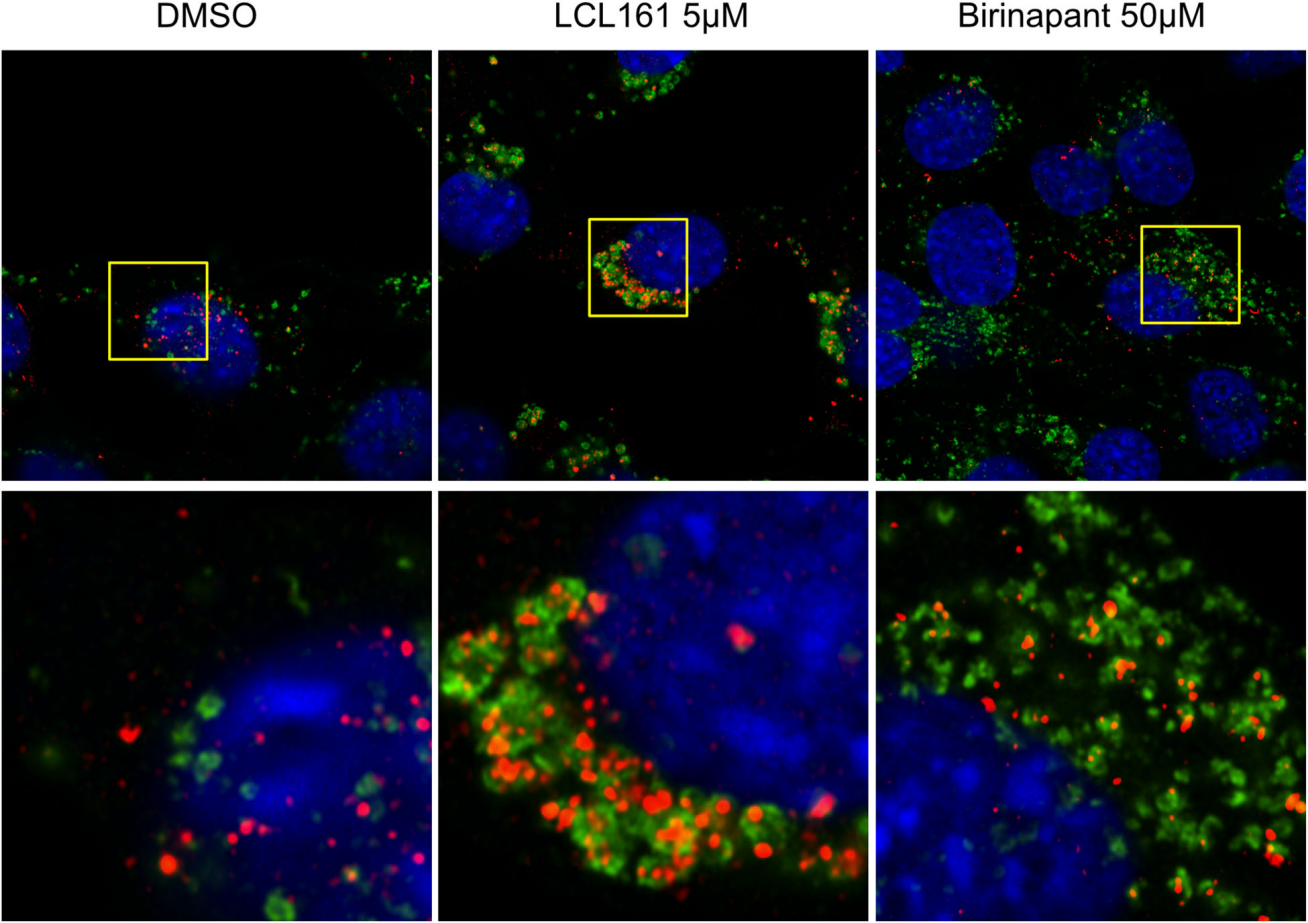
Endogenous LC3 accumulates around lysosomes. Wild type MEFs were treated with LCL161 (5 µM) or birinapant (50 µM) or DMSO as a control for 6 h. Cells were fixed and stained with antibodies against LAMP2 (red channel) and LC3 (green channel). Cells were imaged by confocal microscopy

Electron Microscopy (EM) was performed on LCL161 treated MEFs, as well as thapsigargin treated MEFs and untreated controls. LCL161 treated cells showed a clear increase in vesicular structures containing cellular debris suggesting they are autophagosomes (Fig. 2). The same was seen for thapsigargin treatment. Of note is that electron dense lysosomes cluster in regions adjacent to autophagosomes, but do not appear to be fused with the autophagosomes (Fig. 2). Immunofluoresence was also perfomed on wt MEFs treated with LCL161 or birinapant to confirm that endogenous LC3 also shows a similar accumulation after IAP antagonism (Fig. 3). There is a striking accumulation of autophagosomes, in the perinuclear region. Surprisingly these autophagosomes appear to surround lysosomes (LAMP2^+^ structures) as opposed to co-localising with them which is similar to the non-fused, but associated, autophagosomes and lysosomes seen by EM (Fig. 2) and is indicative of recruitment of autophagosomes to lysosomes, but failure to fuse. The same effect was seen with birinapant but to a lesser extent than with LCL161.

### IAP antagonists block autophagic flux

Blocking fusion of autophagosomes with lysosomes hinders autophagic flux. Autophagic flux can be analysed by preventing formation of new autophagosomes and chasing preformed autophagosomes through to their fusion with lysosomes. Inhibition of PI3K activity blocks the early stages of autophagosome formation, but allows existing autophagosomes to continue through autophagic flux, and be degraded by lysosomes. In cells expressing mCherry-GFP-LC3b this leads to the loss in the GFP signal^29^.

**Fig. 4:**
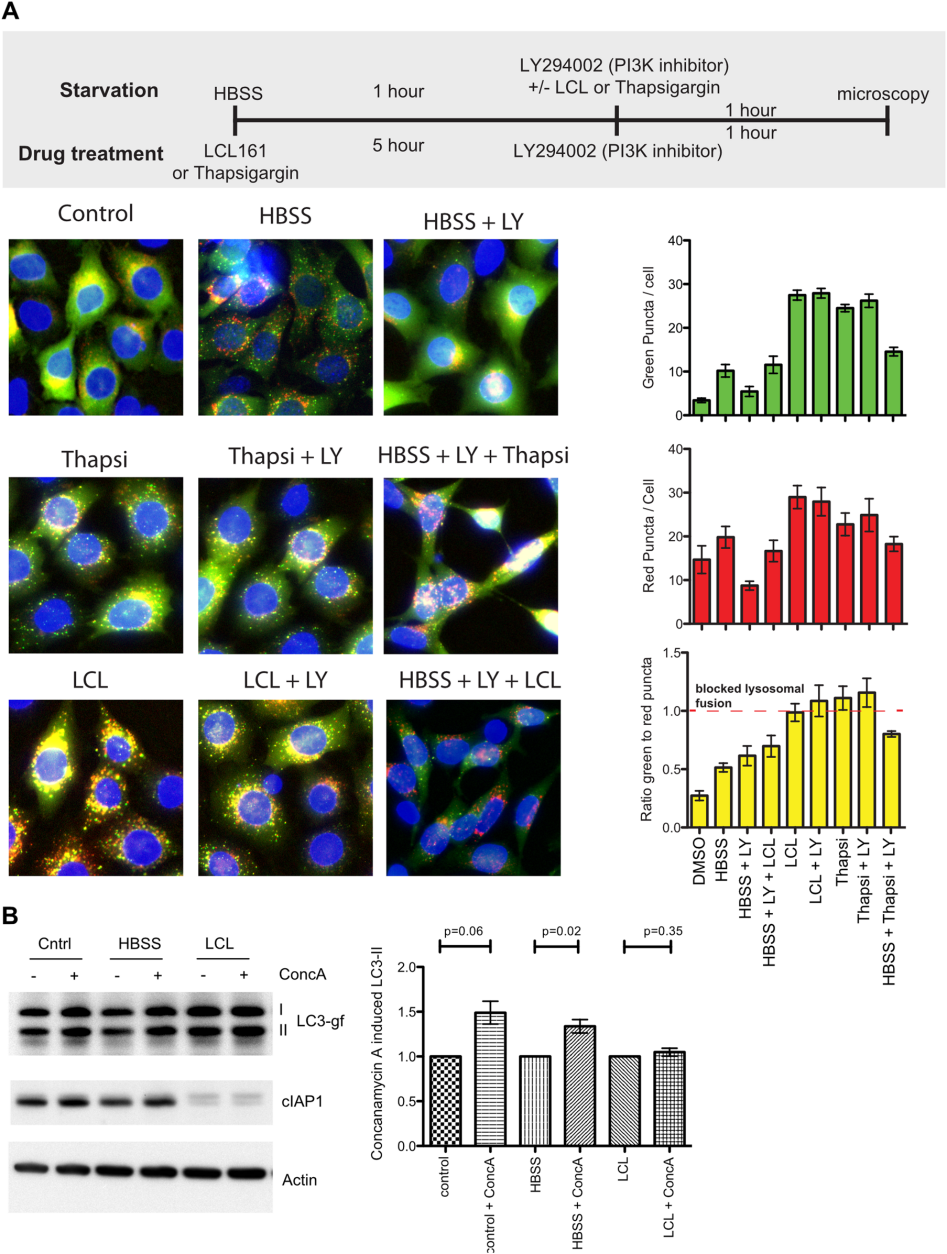
IAP antagonism blocks autophagic flux. **a** MEFs expressing mcherry-gfp-lc3b were treated as shown in the timeline by either starving cells in HBSS for 1 h or treating with thapsigragin (3 μM) or LCL161 (5 μM) for 5 h. LY294002 (20 μM) was then added for 1 h either alone or, in the HBSS starved conditions, thapsigargin or LCL161 were also added together with LY294002. Cells were imaged and GFP + and mCherry + puncta /cell counted. Graphs show means error bars show SEM of at least 3 experiments. **b** MEFs expressing mCherry-GFP-LC3b were either left untreated, starved in HBSS for 2 h or treated with LCL161 (0.5 μM) for 6 h. In each condition cells were also treated with or without concanamycin A (2 nM) for the final hour. Cells were lysed and levels of mCherry-GFP-LC3B detected by western blot. For each condition the amount of mCherry-GFP-LC3BII was normalised to actin levels in the non concanamycin A treated sample. The graph shows the mean fold change in mCherry-GFP-LC3BII from at least 3 independent experiments and error bars show SEM. P values were calculated using *T*-test in Prism software

To analyse if IAP antagonists can block fusion with lysosomes, cells were first starved in HBSS and treated with different combinations of PI3K inhibitor and/or IAP antagonist or thapsigargin. When cells were first starved by incubation in HBSS for 2 h, there was, as expected, a substantial increase in the number of GFP + puncta, while the number of mCherry + puncta remained grossly similar (Fig. 4a). Addition of LY294002 (LY), a PI3K inhibitor known to block autophagy, for 1 h caused the number of GFP + puncta to return to control levels, showing that the existing autophagosomes fused with lysosomes and lost their GFP fluorescence. Addition of LCL161 with LY reversed this GFP + puncta loss, as did addition of thapsigargin. Conversely, addition of LY to cells pre-treated with LCL161 or thapsigargin failed to reduce the number of autophagosomes which had accumulated (Fig. 4a) confirming that pre-existing autophagosomes did not chase through to fusion with lysosomes. These data show that LCL and thapsigargin can both block the turnover of existing autophagosomes that have been triggered to form by starvation. These experiments were also performed with 0.5 μM LCL161 with similar results although reduced in degree (data not shown).

Autophagic flux was further analysed by measuring induction of LC3-II in response to concanamycin A treatment. Concanamycin A, as described above, blocks the last stages of autophagy by blocking acidification of lysosomes. Addition of concanamycin A therefor leads to increased LC3-II levels when autophagy is induced for example by starvation, but shows no increase in LC3-II levels if the late stages of autophagy are blocked, due to the redundancy in effect. We therefore treated MEFs expressing mCherry-GFP-LC3 with either HBSS to induce starvation, or with LCL161. Cells were treated with concanamycin A and analysed by Western blot for levels of mCherry-GFP-LC3-II. There was a clear concanamycin A induced increase in LC3-II in both control and HBSS treated cells, however concanamycin A failed to increase LC3-II in LCL161 treated cells (Fig. 4). Similar results were seen using birinapant (data not shown). These results again argue that IAP antagonism blocks autophagy autophagosome–lysosome fusion stage.

### cIAP2 and XIAP but not cIAP1 regulate autophagic flux

**Fig. 5:**
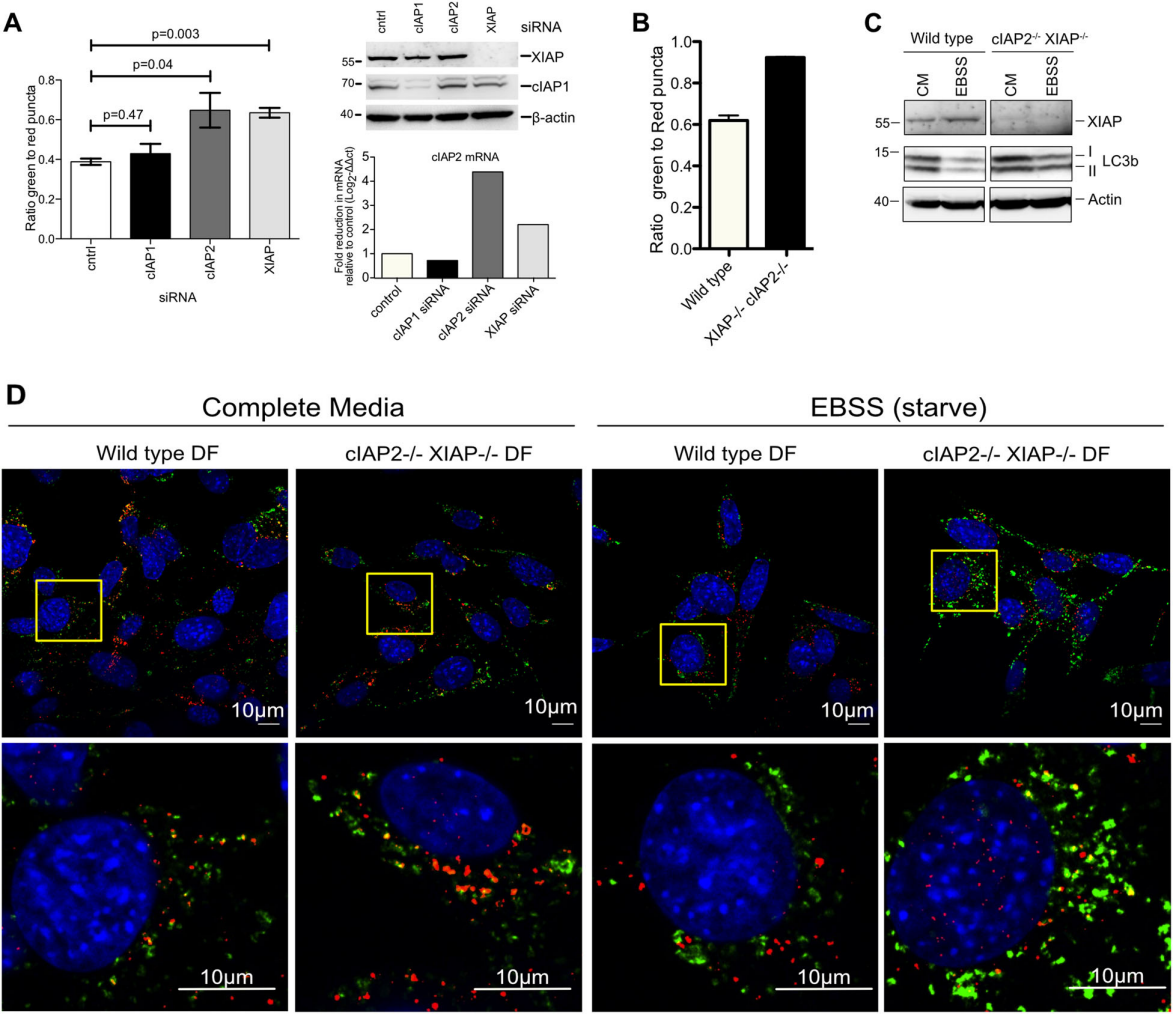
cIAP2 and XIAP regulate autophagosome fusion, but not cIAP1. **a** MEFs expressing mCherry-GFP-LC3b were transfected with siRNA against either cIAP1, cIAP2 or XIAP. Cells were analysed with the microscope and the number of mCherry+, and GFP + puncta/cell were calculated and the ratio of GFP + /mCherry + puncta is indicated. Shown are the means and the error bars represent the SEM of at least three independent experiments. Westerns show efficient knockdown of cIAP1 and XIAP expression. cIAP2 siRNA efficiency was determined by real time PCR as shown in the graph below the westerns. **b** Wild type and cIAP2^−/−^ XIAP^−/−^ dermal fibroblasts expressing mCherry-GFP-LC3b were treated analysed on the microscope and mCherry+, and GFP + puncta/cell were calculated. The ratio of GFP+/mCherry + puncta is indicated. **c** Wild type and cIAP2^−/−^ XIAP^−/−^ dermal fibroblasts were left in complete media (CM) or starved for 2 h in EBSS. Cells were lysed and proteins analysed by western blot for XIAP, LC3, and Actin. cIAP2^−/−^ was confirmed by PCR due to lack of effective antibodies for mouse cIAP2 (see supplemental Fig. 2). **d** Immunofluoresence showing accumulation of LC3 in starved cIAP2^−/−^ XIAP^−/−^ dermal fibroblasts. Wild type and cIAP2^−/−^ XIAP^−/−^ dermal fibroblasts were incubated in complete media or starved in EBSS for 2 h, then fixed and stained for LC3 (green channel) and LAMP2 (red channel). Nuclei are stained blue with Hoechst. Shown in upper panels are overviews. Lower panels show zoomed in regions indicated in the upper panels

LCL161 and birinapant both target cIAP1 and cIAP2 with higher affinity than XIAP. The increase in severity of the phenotype in response to higher doses of LCL161 suggests that XIAP may play a role. To identify which IAPs regulate autophagy and to confirm that this phenotype was specific to IAP antagonism and not an off target effect of the IAP antagonist drugs, siRNA knockdown was used to silence expression of cIAP1, cIAP2 or XIAP. Efficient silencing was achieved for each gene (Fig. 5a). cIAP2 loss was confirmed using qPCR, cIAP1 and XIAP loss were determined by western blot. Loss of cIAP2 or XIAP increased GFP + /mCherry + ratio closer to 1, similar to IAP antagonist treatment at lower doses (0.5 µM) (Fig. 5). Surprisingly however, siRNA against cIAP1 had no significant effect on the GFP + /mCherry + ratio suggesting that build-up of GFP + autophagosomes due to IAP antagonism is likely a result of cIAP2 and XIAP antagonism. We were unable to suppress expression of both cIAP2 and XIAP simultaneously using siRNA in MEFs; at the concentrations required the transfection reagents alone induced build-up of autophagosomes, something that has previously been reported^30^. However, given the higher affinity of birinapant and LCL161 for cIAP1 and cIAP2 over XIAP, the effect seen at low doses is likely mostly due to cIAP2 inhibition.

To determine if loss of both cIAP2 and XIAP together could replicate inhibition of autophagosome–lysosome fusion seen with higher doses of IAP antagonist drugs, cIAP1^fl/fl^ cIAP2^−/−^ XIAP^−/−^ dermal fibroblasts were infected with lentivirus expressing mCherry-GFP-LC3, and the ratio of GFP + /mCherry + vesicles quantified. Genotyping of the cells confirmed knockout of cIAP2 and XIAP (Supplementary Fig. 2). The cIAP1^fl/fl^ cIAP2^−/−^ XIAP^−/−^ cells showed a GFP + /mCherry + ratio close to 1 without any treatment (Fig. 5b). Additionally, cIAP1^fl/fl^ cIAP2^−/−^ XIAP^−/−^ cells have increased endogenous LC3 levels (Fig. 5c). Starvation reduced LC3-II levels in wild type cells, probably due to a high rate of flux, but this was less so for cIAP1^fl/fl^ cIAP2^−/−^ XIAP^−/−^ cells, indicating a reduced rate of flux (Fig. 5c). Immunofluorescence against Lamp2 and LC3 showed dramatic accumulation of LC3 in the starved cIAP1^fl/fl^ cIAP2^−/−^ XIAP^−/−^ cells compared to the wild type (Fig. 5d). At steady state, while there is some accumulation of autophagosomes, the difference is not as clear, suggesting adaptation to loss of cIAP2 and XIAP (Fig. 5d). Loss of both cIAP2 and XIAP together therefore inhibits autophagosome–lysosome fusion causing reduced flux through the autophagy pathway

### IAP antagonists block turnover of long-lived proteins

Basal autophagic activity is responsible for degradation of long-lived proteins that are not normally turned over by proteasomal degradation. To determine if IAP inhibition could block turnover of long-lived proteins, wild type and Atg5^−/−^ MEFs were labelled using click-iT chemistry with L-Azidohomoalanine (AHA). AHA is incorporated in place of methionine when cells are cultured in methionine free media. The degradation of long-lived proteins can then be monitored by labelling with a fluorophore and monitoring fluorescence by flow cytometry^31^.

**Fig. 6:**
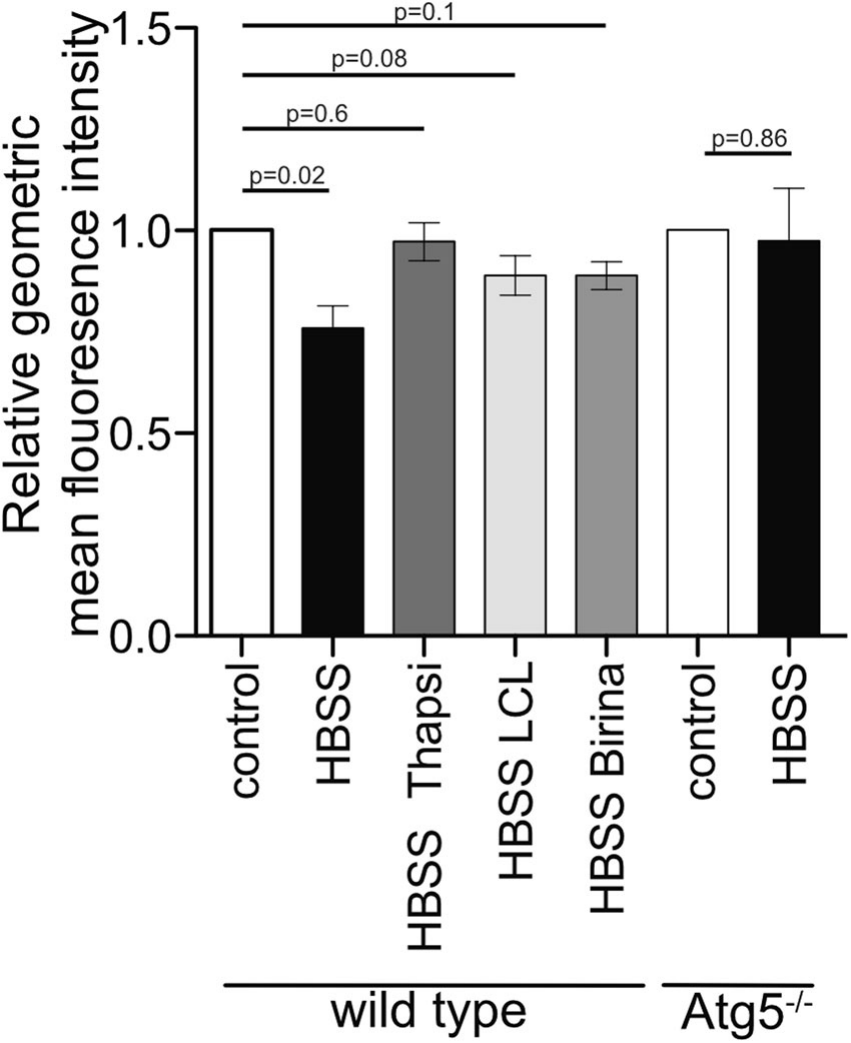
IAP antagonism blocks turnover of long lived proteins. Wild type or Atg5^−/−^ MEFs were labelled overnight with AHA as described in methods. Cells were starved in HBSS or treated with HBSS and LCL161 (0.5 μM), birinapant (0.5 μM) or thapsigargin (3 μM) for three hours. Click it chemistry was used to label proteins and the geometric mean fluorescence intensity was measured for each condition. Shown are the means of at least three independent experiments and error bars show SEM

Starvation in HBSS for three hours resulted in a decrease in fluorescence indicating a reduction of existing stained proteins (Fig. 6). The same treatment in ATG5^−/−^ MEFs showed no significant reduction in fluorescence confirming the reduction observed is specific to autophagy mediated degradation. Co-incubation of wild type cells with HBSS and thapsigargin also completely blocked the decrease in labelled protein, showing that blocking fusion of autophagosomes and lysosomes also blocks protein turnover. Co-incubation with HBSS and LCL161 or birinapant also blocked turnover of long-lived proteins although incompletely, presumably because at these lower doses XIAP is not targeted and only the loss of cIAP2-function was observed (Fig. 6).

### IAPs do not regulate endocytosis

**Fig. 7:**
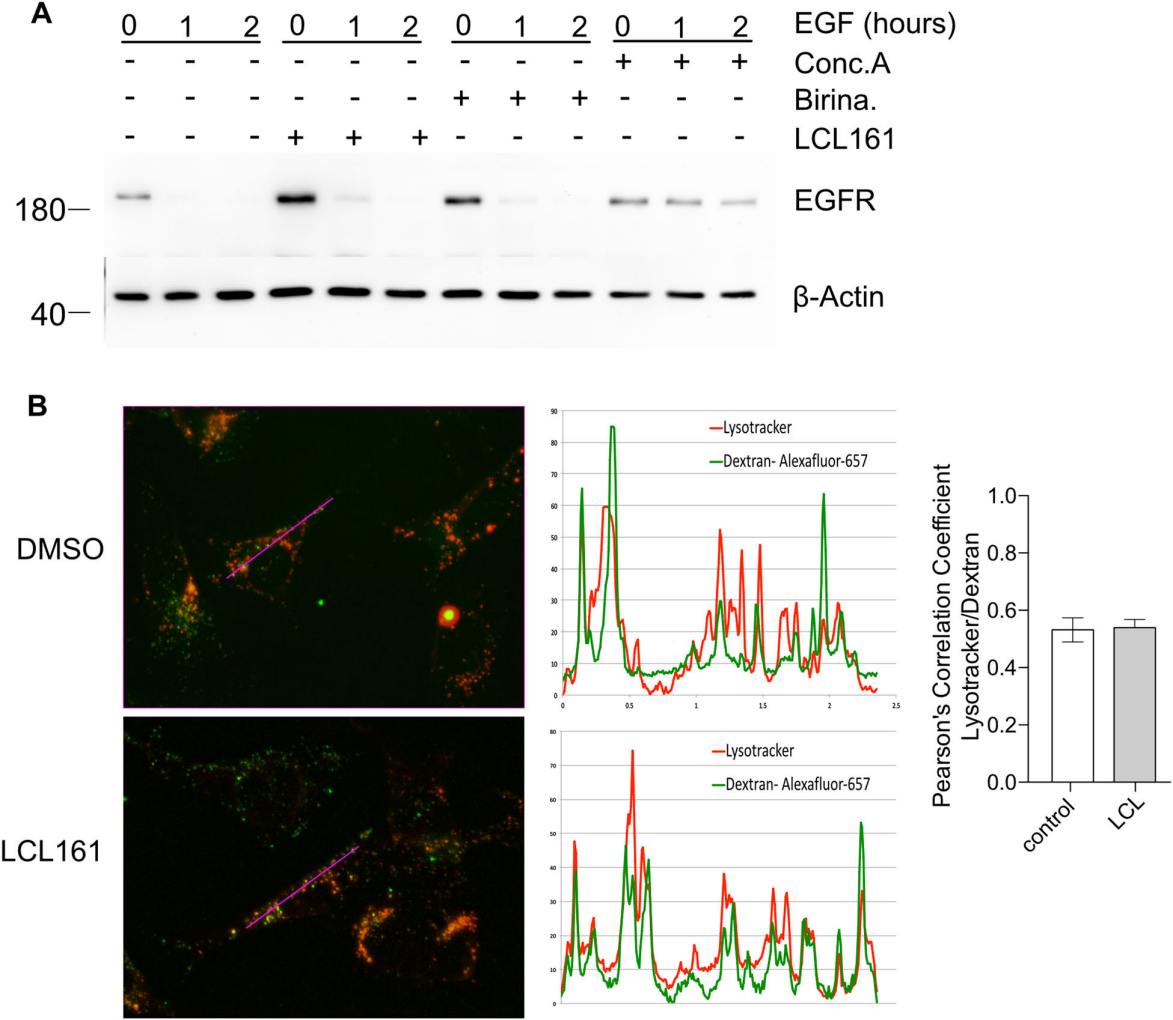
IAP antagonism does not block endocytosis. **a** Wild type MEFs were grown overnight in 0.5% serum. Cells were either left untreated as a control or treated with LCL161 (0.5 μM), birinapant (0.5 μM) for 4 h, or concanamycin A (2 nM) for 1 h before treatment with 100 ng/ml EGF in the presence of 10 μg/ml cycloheximide for 0, 1 or 2 h. Cells were lysed and levels of EGFR were analysed by western blot. **b** Wild type MEFs were treated with LCL161 (0.5 μM) or not for 4 h. Alexafluor 647-dextran (100 μg/mL) was added for 30 mins and then cells were washed and grown in normal media for a further 2 h. The final 30 min cells were stained with lysotracker (100 nM). Cells were fixed and analysed on the fluorescence microscope. Shown are representative images and line profile of lysotracker and dextran Alexafluor 647. Experiments were performed at least 3 times. Pearsons correlation coefficients are shown in the right panel. Error bars represent the SEM from at least three experiments

Autophagosomes use some of the same machinery for lysosomal fusion as the endocytic trafficking system including SNAREs, Rab7 and the class C/HOPS tethering complex^29, 32, 33^. Blockage of autophagosome–lysosome fusion may represent a more general defect in endocytic membrane fusion caused by IAP antagonists. To test this, we monitored internalisation and degradation of EGFR, which upon activation is degraded in lysosomes after endosomal trafficking. There was no change in EGFR degradation rate in LCL161 treated cells while concanamycin A treated cells showed a near complete loss of degradation (Fig. 7a). Additionally we analysed fluid phase endocytosis using uptake of fluorescently labelled dextran and trafficking of labelled endosomes to lysosomes by co-staining with lysotracker. No difference could be detected in the amount of dextran-labelled endosomes co-localising with lysosomes in treated and control cells (Fig. 7b). This was confirmed by Pearson’s correlation coefficient analysis, which also clearly showed that the degree of co-localisation is not altered significantly by IAP antagonism (Fig. 7b). Together these results clearly show that IAP antagonism has no effect on the endosomal pathway but instead specifically affects the autophagosomal system.

### Mitophagy and xenophagy are blocked in IAP antagonist-treated cells

**Fig. 8:**
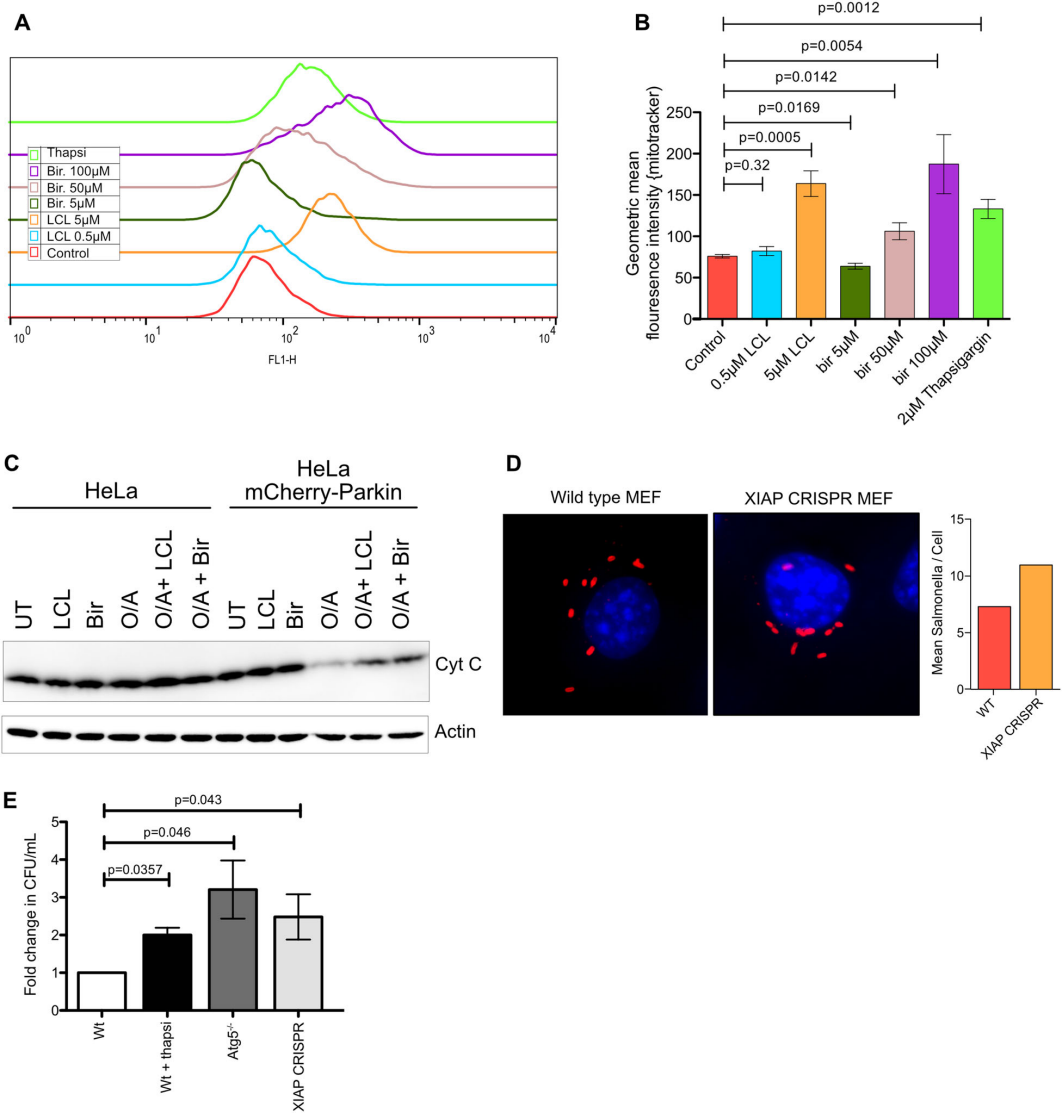
IAP antagonism blocks mitophagy and xenophagy. Wild type MEFs were treated overnight with the indicated concentrations of LCL161, birinapant or thapsigargin. Cells were stained with mitotracker green for 30 min and fluorescence intensity quantified using flow cytometry. **a** representative histograms showing increases in mitotracker signal in IAP antagonist treated cells. **b** quantification of mitotracker staining. Shown are the means of the geometric mean fluorescence intensity. Error bars are SEM from at least 3 independent experiments. *P* values were calculated using *t* tests in Prism software. **c** IAP antagonism can block mitophagy. Wild type HeLa cells or HeLa cells expressing mCherry-Parkin were treated with either LCL161 (5 µM), birinapant (50 µM) or oligomycin (1 µM) and Antimycin A (1 µM) (O/A) alone or in combination as indicated. LCL161 or birinapant were added 4 h prior to Oligomycin and Antimycin A. Cells were incubated for 16 h before cells were harvested and lysates run on SDS-PAGE and levels of cytochrome C and β-Actin analysed by western. **d** Immunofluoresence and quantification of *Salmonella* Typhimurium infection in wild type and XIAP CRISPR MEFs. MEFs of the indicated genotypes were infected as described in methods, fixed and stained for *Salmonella* Typhimurium LPS. Shown are representative images. The mean number of *Salmonella* Typhimurium /cell is shown from two experiments. **e** Xenophagy of *Salmonella* Typhimurium is impaired in XIAP deficient cells. Wild type, Atg5^−/−^ and XIAP^−/−^-CRISPR cells were infected with *Salmonella* Typhimurium and incubated for 5 h. CFU/mL of internalised bacteria was then calculated. Thapsigargin was used where indicted to block autophagosome–lysosome fusion by treating with 3 µM thapsigargin for 1 h before and then throughout the infection protocol. Error bars are SEM from at least 3 independent experiments. *P* values were calculated using *T* tests in Prism software

One consequence defective autophagy is the accumulation of mitochondria due to failure loss of mitophagy. We examined the amount of mitochondria in MEFs treated with LCL161 or thapsigargin by staining with Mitotracker Green (MG). High dose (5 µM) LCL161 and thapsigargin treated cells showed a clear increase in MG signal, while low doses (0.5 μM) showed no significant increase (Fig. 8a, b). Birinapant also showed an increase in mitochondrial mass only at the high doses that fully block fusion (Fig. 8a, b). To confirm that mitophagy is impaired we turned to HeLa cells overexpressing Parkin as a well-established model for inducing mitophagy. HeLa cells do not express endogenous Parkin, a ubiquitin ligase that ubiquitylates damaged mitochondria and induces their clearance by mitophagy^34^. We treated HeLa cells without Parkin and cells overexpressing mCherry-Parkin with Oligomycin and Antimycin A to induce mitochondrial damage either with or without LCL161 or birinapant for 16 h and examined the levels of cytochrome C as a marker of mitochondria. There was a decrease in cytochrome C levels with the oligomycin and Antimycin A treatment only in the Parkin over-expressing cells (Fig. 8c). Addition of LCL161 restored the levels of cytochrome C to some extent, however not completely (Fig. 8c). MEFs were also induced to over-express Parkin and treated in the same way, but in our hands with all mitochondrial proteins tested we also saw degradation in ATG5^−/−^ MEFs and also in non-Parkin over-expressing cells (data not shown), suggesting that the degradation is not mitophagy in the MEFs. Similar results have been published previously indicating that there are few reliable markers for mitophagy in MEFs^35^. Therefor, not only is mitophagy inhibited in IAP antagonist treated cells, but IAP antagonism also blocks or at least slows the degradation of mitochondria by mitophagy in human cells too.

Xenophagy, which is conceptually similar to mitophagy as it involves the tagging of intracellular bacteria with ubiquitin followed by engulfment by autophagosomes and degradation, is an early step in the recognition of a number of pathogenic bacteria. *Salmonella* have been used extensively as a model to study xenophagy and there is significantly more bacterial survival in autophagy deficient cells^36^. A block in mitophagy suggests loss of XIAP could also lead to a deficiency in xenophagy. To test this, XIAP was knocked out in MEFs using CRISPR-Cas9. Cells infected under these conditions were fixed and stained using an antibody against *S*. Typhimurium LPS and the number of bacteria in each cell counted, revealing more salmonella in the XIAP CRISPR cells than in the wild type (Fig. 8d). This was then confirmed by lysing cells and calculating the colony forming units (CFU) (Fig. 8e). Most studies analysing xenophagy have used treatments or mutants that block the early stages of autophagosome formation. To the best of our knowledge little is known about the effect of blocking fusion of autophagosomes with lysosomes on xenophagy. As a model for blockage of the fusion of autophagosomes with lysosomes, cells were also treated with thapsigargin. Following 5 h of infection, there is consistently more surviving bacteria in ATG5^−/−^ cells than in wild type as previously reported. Thapsigargin treatment also resulted in accumulation of viable *S*. Typhimurium, as did knockout of XIAP in the XIAP-CRISPR cells (Fig. 8e). Both thapsigargin and XIAP deficiency result in increased *S*. Typhimurium survival to a similar extent to Atg5 deficiency, supporting that Autophagy inhibition is involved in all conditions.

## Discussion

While we have demonstrated that cIAP2 and XIAP promote autophagosome-lysosome fusion, the mechanism is still not clear. Ubiquitylation regulates many steps in vesicle trafficking^37^ and it is likely that cIAP2 and XIAP are regulating specific components of the autophagosome–lysosome fusion machinery via their ubiquitin ligase activity. The observation that endocytosis is also not affected by IAP inhibition suggests ubiquitylation of some target on the autophagosomes themselves is likely, however we cannot at this stage rule out that some other function such as scaffolding may also be playing a role.

One interesting aspect of these results is that we have identified cIAP2 and XIAP as playing redundant roles in regulating autophagy in MEFs. XIAP depletion alone in humans leads to disease but XIAP^−/−^ mice are in most aspects normal, although they can be induced to develop disease similar to XLP-2 in humans by infection with a herpes virus, MHV-68^38^. One might expect that if there was an absolute requirement for XIAP or cIAP2 in autophagy that mice deficient for either alone would present with phenotypes associated with defects in autophagy, but loss of both seems to be required before spontaneous inflammation is seen^2^. This may be partly explained by the overlapping functions seen for cIAP2 and XIAP in this study and may explain why XIAP deficient mice do not develop disease like humans do.

Relatively little is known about the substrates of XIAP, however ubiquitylation of RIPK2 at NOD2 receptors was shown to be required for NOD2 activation of NF-κB^13^. Loss of NF-κB signalling results in a failure to up-regulate cytokines in response to NOD2 signalling and this link to NOD2 signalling is thought to be the cause of Crohn’s disease in XIAP deficient patients. While this molecular connection between NOD2 and XIAP may contribute to disease progression, NOD2 mutation does not lead to the multiple other conditions that XIAP deficiency causes^39^, suggesting that the story is not so simple. There are numerous genes that are involved in autophagy, including ATG16L1, NDP52, IRGM, and LRRK2 that have been found to be mutated in Crohn’s Disease patients; a number of these mutations also manifest with multiple clinical presentations. Recently, Niemann–Pick disease type C (NPC), a lysosomal storage disorder in which the NPC1 gene coding for a lysosomal protein involved in lipid transport is mutated, was also linked to early onset Crohn’s disease. Loss of NPC1 function was also shown to be linked to defective autophagy resulting in increased survival of intracellular *Salmonella* and adherent-invasive *E. coli*^40^. Importantly, this study showed that NOD2 induced NF-κB signalling is normal in NPC1 mutated cells and enforced autophagy induction could bypass the xenophagy defect and reduce intracellular bacterial survival. Similarly impaired xenophagy has also been seen in macrophages from patients with NOD2 and XIAP mutations. We observed defects in xenophagy of *S*. Typhimurium in XIAP deficient fibroblasts. Such defects in xenophagy may also contribute to accumulation of invasive bacteria in XIAP deficient humans, again increasing the burden of bacteria that needs to be dealt with by the immune system and resulting in overactive inflammation. Additionally we observed an accumulation of mitochondria in cells treated with IAP antagonists at doses high enough to block XIAP, as well as cIAP1 and cIAP2, suggesting that the loss of IAPs also causes defects in mitophagy. Mitophagy is important for clearing old or damaged mitochondria and defects in mitophagy have been linked to excessive ROS production and excessive inflammasome activation^41^. Mitophagy is in many ways mechanistically analogous to xenophagy and defects therein may also contribute to inflammation in XIAP deficient patients. This suggests that the driving mechanism behind multiple genetic mutations causing Crohn’s Disease may be a loss of autophagy, and supports a role for XIAP in regulating this process.

Many questions remain including dissection of the mechanism behind cIAP2 and XIAPs regulation of autophagosome–lysosome fusion, as well as the degree to which autophagy defects contribute to inflammation induced by IAP loss. It will be interesting to see if IAPs or their substrates are also involved in other autophagy related diseases such as protein misfolding diseases and or lysosomal storage disorders.

## Methods

### Reagents

LCL161 and birinapant were purchased from ApexBio. The following antibodies were used; EGFR (PA1-1110—Thermo fisher), XIAP (MAB822—R&D Systems), cIAP1 (was a kind gift from John Silke—WEHI Melbourne Australia), LC3b (nb100–2220—Novus), LAMP2 (ab13524—Abcam). EGF was purchased from Biolegend (catalogue No. 713108). Alexafluor 647 Dextran MW10,000 (catalogue No. D-22914), Lysotracker Red DND-99 (catalogue No. L7528) and Mitotracker Green (catalogue No. M7514) were purchased from Thermo Fisher Scientific. Concanamycin A (catalogue No. Cay-11050-25) was purchased from Cayman Chemical.

### Cell culture

All cells were grown in DMEM High glucose (Gibco) with 10% FCS and 1% Pen/Strep in 5% CO_2_ at 37 °C unless otherwise stated.

### Fluorescence microscopy of LC3

MEFs of the indicated genotypes were infected with pBABE-puro-mCherry-EGFP-LC3B (pBABE-puro-mCherry-EGFP-LC3B was a gift from Jayanta Debnath (Addgene plasmid # 22418)). Cells were selected in Puromycin 4 μg/ml. The selected cells were seeded at 50,000 cells/well of an 8 chamber μ-slide (Ibidi) and allowed to plate down overnight. Cells were typically treated for 6 h with the indicated concentrations of LCL161, birinapant or thapsigargin (3 μM). Cells were treated for 30 min with Hoechst to stain nuclei. Cells were imaged with a Keyence BZ9000 fluorescence microscope taking Z-stacks to and using the “full focus” function in the analysis software to make a single layer image from full focused features in each layer of the Z-stack. These images were used to count the number of GFP + and mCherry + puncta per cell in image J using the analyse particles function to count nuclei and find maxima function to count puncta. The ratio of mCherry + /GFP + puncta/cell was calculated from these measurements.

For Immunofluorescence of LC3 and LAMP2, Wild type MEFs were seeded in 8 well µ-slides (Ibidi) at 50,000 cells/well and incubated overnight. Cells were treated with IAP antagonists as indicated and cells were washed twice in warm PBS. Cell were fixed in 4% PFA in PBS for 10 min followed by washing three times in PBS. Cells were subsequently permeabilized in PBS with 50 µg/ml Digitonin for 5 min followed by washing three times in PBS then blocking with 3% BSA in PBS for 30 min. Antibodies against LC3 and Lamp2 were added 1:200 for 1 h in BSA 3% in a humidified chamber at 37 C. Cells were washed three times in PBS followed by incubating with appropriate secondary antibodies at 1:500 in PBS 3% BSA for 1 h followed by washing 5 times in PBS. Images were taken using a Zeiss LSM 880 with an Airyscan confocal microscope at a 63 × magnification (oil immersion) and analysed with Zen (Zeiss) software.

### siRNA treatment

MEFs were seeded at 150,000 cells/well of a 6 well plate. The next day cells were washed with PBS and media without antibiotics was added. Cells were transfected with siRNA’s complexed with Lipofectamine RNAiMAX according to manufacturers instructions. Briefly, 7.5 µL of 20 µM stock stealth siRNA oligo was added to 150 µL of optimem. Nine microlitre of RNAiMAX was added to another 150 µL of optimum. The tubes were mixed together before incubating at room temperature for 5 min. 250 µL of this was then added to the cells and incubated overnight. Cells were seeded at 50,000 cells/well of an 8 chamber μ-slide (Ibidi) and allowed to plate down overnight before microscopy was performed as described above. The following stealth siRNAs were used from Thermo Fisher Scientific; cIAP1 (MSS273215), cIAP2 (MSS202113) and XIAP (MSS202115). Activity of each siRNA was confirmed using qPCR with SYBR Select Master Mix (applied biosystems # 4472908) and the following primers: cIAP1 (Forward 5′-GAAGAAAATGCTGACCCTACAGA-3′, Reverse 5′-CATGACGACATCTTCCGA-3′), cIAP2 (Forward 5′- TCGATGCAGAAGACGAGA-3′ Reverse 5′-TTTGTTCTTCCGGATTAGTGC-3′, XIAP (Forward 5′-GCTTGCAAGAGCTGGATTTT-3′, Reverse 5′-TGGCTTCCAATCCGTGAG-3′). Actin was used as a reference gene. PCR was done in 384 well plates in a 7900HT Fast Real-Time PCR System.

### Long-lived protein degradation assay

Cells were grown to ~70–80% confluency in a 6-well plate then washed with warm PBS and cultured in L-methionine-free DMEM (cat. no. 21013-024, Gibco) for 30–60 min to deplete the intracellular methionine reserves. Following methionine depletion, the cells were labelled with 25 μM AHA in 10% dialysed FBS DMEM (methionine-free) for 18 h. Dialysed FBS was made by dialyzing against PBS with Slide-A-Lyzer mini dialysis devices 3.5 k MWCO (Pierce—cat. No. 88403) overnight. After labelling, the cells were washed with PBS and cultured in regular DMEM containing 10 × L-methionine (2 mM) for 2 h to chase out short-lived proteins. LCL161 (0.5 μM), thapsigargin (3 μM), or birinapant (0.5 μM) were added at this stage. Cells were washed in PBS and then cultured for a further 3 h either in full media or HBSS containing the indicated drugs. Cells were washed 2× in PBS and then harvested and fixed in 4% formaldehyde in PBS for 15 min at room temperature. After fixation, the cells are washed twice with 3% BSA in PBS. Cells were permeabilized with 0.5% Triton X-100 in PBS for 20 min at room temperature. Cells were resuspended in PBS and store at 4 °C for detection of the corresponding alkyne-tagged detection molecule.

Cells from the last step above were washed with 3% BSA in PBS. 100 μL Click-iT® reaction cocktail was added to each sample. Cells were incubated for 30 min at room temperature in the dark. Cells were washed once with 3% BSA in PBS before being analysed using flow cytometry.

### EGFR degradation assay

A total of 500,000 cells/well were seeded in the morning and let to plate down. In the evening, media was exchanged for DMEM 0.5% FCS and cells incubated overnight. The next day, cells were pre-treated for 4 h with IAP antagonists birinapant (0.5 μM) or LCL161 (0.5 μM) or for 1 h with concanamycin A (2 nM). Media was then exchanged with serum free media alone or containing the drugs. Cells were treated with EGF (100 ng/ml) and cycloheximide (10 μg/ml) for 0, 1 or 2 h. Cells were lysed on ice in RIPA buffer and 50 µg/ml protein per lane was run on 10% SDS-PAGE before transferring to nitrocellulose membranes and western blotting for EGFR

### Assay of endocytosis and fusion with lysosomes

30,000 cells/well were seeded in 8 well microscope culture slides (Ibidi) in 300–500 µL media. The next day cells were treated with DMSO, LCL161 0.5 μM or birinapant (0.5 μM) for 6 h. 2.5 h before end of drug treatments, Dextran Alexafluor 647 was added to a final of concentration of 100 µg/ml. Cells were incubated for 30 min at 37 °C. Cells were washed 2× in warm PBS. Fresh media containing LCL161 or birinapant was added and cells were incubated for a further 2 h. Lysotracker Red (100 nM) was added for the final 30 min. Cells were washed 2× in warm PBS before fixation in 4% paraformaldehyde (PFA) for 20 min at room temperature. Cells were washed 2× in PBS and stored at 4 °C until images were taken on a Keyence BZ9000 fluorescence microscope. Hoechst was added 30 min prior to imaging. Pearsons correlation coefficients were calculated using the FIJI distribution of ImageJ and the Coloc2 plugin.

### Electron microscopy

MEFs were cultured on glass cover slips and fixed for 20 min in 4% PFA plus 1% glutaraldehyde (Roth, Germany) in PBS. After contrastation in 0.5% Osmiumtetroxide (30 min at RT) cells were dehydrated and embedded in epoxy resin (Durcupan, Sigma-Aldrich, Gillingham, UK). Images were taken using a Philips CM 100 transmission electron microscope.

### Mitophagy assays

For mitotracker Green staining, Wild type MEFs were treated overnight with the indicated concentrations of LCL161, birinapant or thapsigargin. Cells were incubated with 100 nM mitotracker Green for 30 min in complete media then washed in PBS and trypsinized then analysed for fluorescence intensity using flow cytometry on a FACS Calibur then analysed using FlowJo software.

For analysis of Parkin dependent mitophagy, HeLa cells were infected with a lentivirus (pXLG3) expressing mCherry-Parkin (human)^42^ or left uninfected. These HeLas were then seeded at 4 × 10^5^ for wild type and 6 × 10^5^ cells/well in 6 well plates, left overnight and then incubated for 4 h with 5 µM LCL161 or 50 µM birinapant followed by addition of Oligomycin (1 µM) (Sigma Aldrich—O4876) and Antimycin A (1 µM) (Sigma Aldrich—A8674) for 16 h. Cells were harvested and lysed in DISC lysis buffer followed by detection of Cytochrome C by western blot. Actin was used as a loading control.

### Xenophagy assays

*S*. Typhimurium used in all analysis was a patient derived strain identified through sera agglutination. *Salmonella* were cultured overnight in LB at 37 °C. On the same day 100,000 cells/well of MEFs were seeded in 12 well plates. The next day, *Salmonella* were diluted 1:33 and incubate without shaking for 3 h at 37 °C. *Salmonella* were harvested by centrifugation and washed with PBS two times before re-suspending in DMEM without any antibiotics. The cells were adjusted to have an OD600 of 0.5 by diluting with DMEM. One hundred microlitre of *Salmonella* culture was added to each well of MEFs and plates spun for 5 min in 37 °C centrifuge at 1800RPM. Cells were incubated for a further 5 min at 37 °C before washing cells twice with PBS at 37 °C. incubate cells at 37 °C in DMEM without antibiotics for 20 min. Cells were incubated in DMEM with gentamicin at 50 μg/ml for 40 min at 37 °C. Cells were changed to DMEM containing 5 μg/ml gentamicin and incubated for 5 h at 37 °C. For CFU calculations cells were washed twice with PBS and then lysed in 1 mL of lysis buffer (1% (v/v) TX-100, 0.1% (w/v) SDS in PBS). Serial dilutions were plated onto LB agar plates and CFU/mL calculated. For immunofluoresence infections were perfomed as above but on either glass coverslips or in 8 chamber microslides (Ibidi) and were also washed twice in PBS and fixed in 100% MeOH (−20 °C) for 20 min at (−20 °C). Cells were stained with an antibody against *S*. Typhimurium LPS (santacruz sc52223) (1:100) in PBS with 3% BSA for 30 min followed by staining with anti mouse IgG-Cy3 (1:500) in PBS 3% BSA. Nuceli were stained with Hoechst. Cells were imaged and the number of bacteria per cell was counted from at least 50 cells for each genotype per experiment.

## Electronic supplementary material

Supplemental Figures

## References

[1] Vince JE (2007). IAP antagonists target cIAP1 to induce TNFalpha-dependent apoptosis. Cell.

[2] Wong WWL (2014). cIAPs and XIAP regulate myelopoiesis through cytokine production in an RIPK1- and RIPK3-dependent manner. Blood.

[3] Vince JE (2012). Inhibitor of apoptosis proteins limit RIP3 kinase-dependent interleukin-1 activation. Immunity.

[4] Weber A (2010). Proapoptotic signalling through Toll-like receptor-3 involves TRIF-dependent activation of caspase-8 and is under the control of inhibitor of apoptosis proteins in melanoma cells. Cell Death Differ..

[5] Vince JE (2008). TWEAK-FN14 signaling induces lysosomal degradation of a cIAP1-TRAF2 complex to sensitize tumor cells to TNFalpha. J. Cell. Biol..

[6] Vallabhapurapu S (2008). Nonredundant and complementary functions of TRAF2 and TRAF3 in a ubiquitination cascade that activates NIK-dependent alternative NF-kappaB signaling. Nat. Immunol..

[7] Zarnegar BJ (2008). Noncanonical NF-kappaB activation requires coordinated assembly of a regulatory complex of the adaptors cIAP1, cIAP2, TRAF2 and TRAF3 and the kinase NIK. Nat. Immunol..

[8] Damgaard RB (2012). The Ubiquitin Ligase XIAP Recruits LUBAC for NOD2 Signaling in Inflammation and Innate Immunity. Mol. Cell..

[9] Aguilar C (2014). Characterization of Crohn disease in X-linked inhibitor of apoptosis–deficient male patients and female symptomatic carriers. J. Allergy Clin. Immunol..

[10] Zeissig Y (2014). XIAP variants in male Crohn’s disease. Gut.

[11] Speckmann C, Ehl S (2014). XIAP deficiency is a mendelian cause of late-onset IBD. Gut.

[12] Rigaud S (2006). XIAP deficiency in humans causes an X-linked lymphoproliferative syndrome. Nature.

[13] Damgaard RB (2013). Disease-causing mutations in the XIAP BIR2 domain impair NOD2-dependent immune signalling. EMBO Mol. Med..

[14] Hampe J (2001). Association between insertion mutation in NOD2 gene and Crohn’s disease in German and British populations. Lancet.

[15] Ogura Y (2001). A frameshift mutation in NOD2 associated with susceptibility to Crohn’s disease. Nature.

[16] Hugot JP (2001). Association of NOD2 leucine-rich repeat variants with susceptibility to Crohn’s disease. Nature.

[17] Hampe J (2007). A genome-wide association scan of nonsynonymous SNPs identifies a susceptibility variant for Crohn disease in ATG16L1. Nat. Genet..

[18] Travassos LH (2010). Nod1 and Nod2 direct autophagy by recruiting ATG16L1 to the plasma membrane at the site of bacterial entry. Nat. Immunol..

[19] Homer CR, Richmond AL, Rebert NA, Achkar JP, McDonald C (2010). ATG16L1 and NOD2 Interact in an autophagy-dependent antibacterial pathway implicated in Crohn’s Disease pathogenesis. Gastroenterology.

[20] Lapaquette P, Bringer MA, Darfeuille-Michaud A (2012). Defects in autophagy favour adherent-invasive Escherichia coli persistence within macrophages leading to increased pro-inflammatory response. Cell. Microbiol..

[21] Ktistakis NT, Tooze SA (2016). Digesting the expanding mechanisms of autophagy. Trends Cell Biol..

[22] Anding AL, Baehrecke EH (2017). Cleaning house: selective autophagy of organelles. Dev. Cell.

[23] Zhangyuan Yin CPDJK (2016). Autophagy: machinery and regulation. Microb. Cell.

[24] Bosch ME, Kielian T (2015). Neuroinflammatory paradigms in lysosomal storage diseases. Front. Neurosci..

[25] Shibutani ST, Saitoh T, Nowag H, Münz C, Yoshimori T (2015). Autophagy and autophagy-related proteins in the immune system. Nat. Immunol..

[26] Agyemang AF, Harrison SR, Siegel RM, McDermott MF (2015). Protein misfolding and dysregulated protein homeostasis in autoinflammatory diseases and beyond. Semin. Immunopathol..

[27] Deretic V (2015). Immunologic manifestations of autophagy. J. Clin. Invest..

[28] Weisberg E (2010). leu2010212a. Leukemia.

[29] Ganley IG, Wong PM, Gammoh N, Jiang X (2011). Distinct autophagosomal-lysosomal fusion mechanism revealed by thapsigargin-induced autophagy arrest. Mol. Cell.

[30] Man N, Chen Y, Zheng F, Zhou W, Wen LP (2014). Induction of genuine autophagy by cationic lipids in mammalian cells. Autophagy.

[31] Zhang J, Wang J, Ng S, Lin Q, Shen HM (2014). Development of a novel method for quantification of autophagic protein degradation by AHA labeling. Autophagy.

[32] Shen HM, Mizushima N (2014). At the end of the autophagic road: an emerging understanding of lysosomal functions in autophagy. Trends Biochem. Sci..

[33] Jiang P (2014). The HOPS complex mediates autophagosome-lysosome fusion through interaction with syntaxin 17. Mol. Biol. Cell..

[34] Narendra D, Tanaka A, Suen DF, Youle RJ (2008). Parkin is recruited selectively to impaired mitochondria and promotes their autophagy. J. Cell. Biol..

[35] Baudot AD, Haller M, Mrschtik M, Tait SWG, Ryan KM (2015). Using enhanced-mitophagy to measure autophagic flux. Methods.

[36] Birmingham CL, Smith AC, Bakowski MA, Yoshimori T, Brumell JH (2006). Autophagy controls Salmonella infection in response to damage to the Salmonella-containing vacuole. J. Biol. Chem..

[37] Erpapazoglou Z, Walker O, Haguenauer-Tsapis R (2014). Versatile roles of K63-linked ubiquitin chains in trafficking. Cells.

[38] Yabal, M. et al. XIAP Restricts TNF- and RIP3-dependent cell death and inflammasome activation. *Cell Rep.* **7**, 1796–1808 (2014) 10.1016/j.celrep.2014.05.008 24882010

[39] Speckmann C (2013). X-linked inhibitor of apoptosis (XIAP) deficiency: the spectrum of presenting manifestations beyond hemophagocytic lymphohistiocytosis. Clin. Immunol..

[40] Schwerd T (2017). Impaired antibacterial autophagy links granulomatous intestinal inflammation in Niemann-Pick disease type C1 and XIAP deficiency with NOD2 variants in Crohn’s disease. Gut.

[41] Zhong Z (2016). NF-κB restricts inflammasome activation via elimination of damaged mitochondria. Cell.

[42] Jimenez Orgaz A (2018). Control of RAB7 activity and localization through the retromer‐TBC1D5 complex enables RAB7‐dependent mitophagy. EMBO J..

