# Supplementary material for: Inhibitor of apoptosis proteins are required for effective fusion of autophagosomes with lysosomes
Source: Cell Death Dis. 2018 May 9;9(5):529. doi: 10.1038/s41419-018-0508-y (PMC5943300; doi:10.1038/s41419-018-0508-y)
Supplement: Supplementary file 1 — Supplemental Figures [file 41419_2018_508_MOESM1_ESM.pdf]

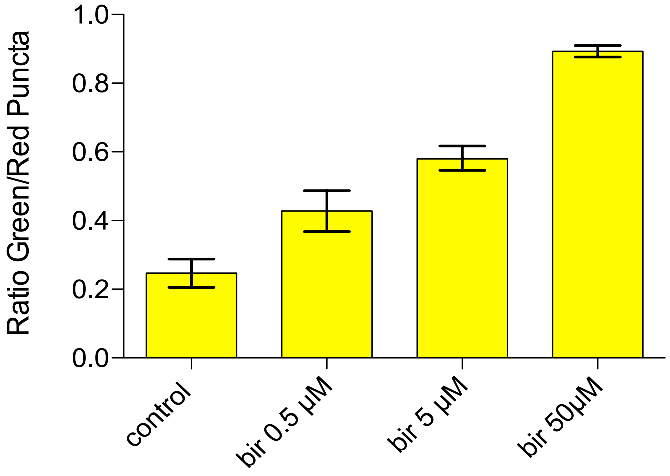
Ratio Green/Red Puncta
1.0
0.8
0.6
0.4
0.2
0.0
control
bir 0.5 µM
bir 5 µM
bir 50µM

**Figure S1**

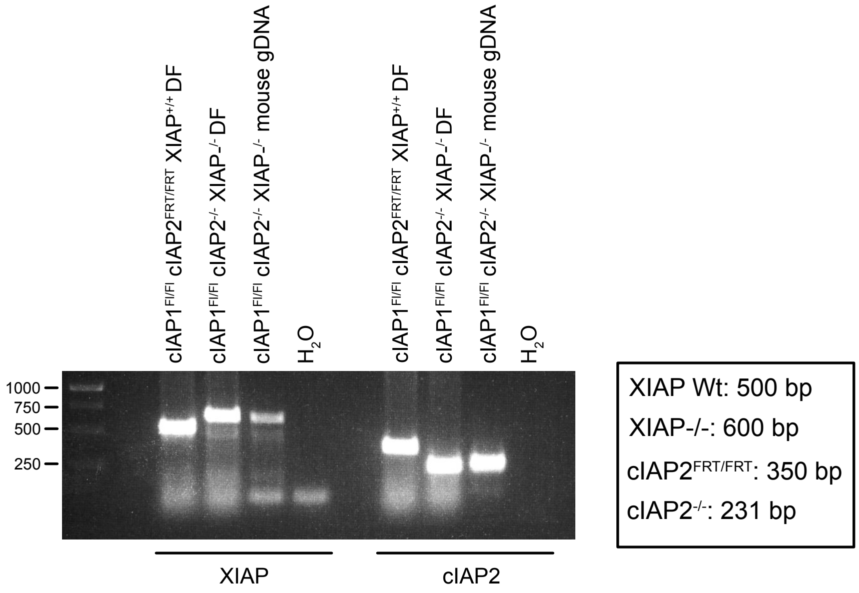

cIAP1$^{Fl/Fl}$ cIAP2$^{FRT/FRT}$ XIAP$^{+/+}$ DF
cIAP1$^{Fl/Fl}$ cIAP2$^{-/-}$ XIAP$^{-/-}$ DF
cIAP1$^{Fl/Fl}$ cIAP2$^{-/-}$ XIAP$^{-/-}$ mouse gDNA
$H_2O$
cIAP1$^{Fl/Fl}$ cIAP2$^{FRT/FRT}$ XIAP$^{+/+}$ DF
cIAP1$^{Fl/Fl}$ cIAP2$^{-/-}$ XIAP$^{-/-}$ DF
cIAP1$^{Fl/Fl}$ cIAP2$^{-/-}$ XIAP$^{-/-}$ mouse gDNA
$H_2O$
1000
750
500
250
XIAP
cIAP2
XIAP Wt: 500 bp
XIAP-/-: 600 bp
cIAP2$^{FRT/FRT}$: 350 bp
cIAP2$^{-/-}$: 231 bp


**Figure S2**

## Supplemental Figure Legends

**Figure S1: Birinapant can block autophagosomal-Lysosomal fusion at high doses.**

Wild type MEFs expressing mcherry-GFP-LC3b were treated with the indicated doses of Birinapant for 6 hours. DNA was stained using Hoechst and live cells were then imaged to visualize mCherry, GFP and Hoechst. The number of GFP positive puncta and mCherry positive puncta were then counted per cell and the ratio of GFP+/mCherry+ puncta calculated. Graphs represent the mean and error bars show SEM of at least 3 experiments.

**Figure S2: Confirmation of genotype of cIAP2$^{-/-}$ XIAP$^{-/-}$ Dermal fibroblasts**

Genomic DNA was harvested from Dermal fibroblasts of the indicated genotypes as well as control DNA form a cIAP2$^{-/-}$ XIAP$^{-/-}$ mouse. PCR was performed to show the correct genotype. Expected sizes of PCR products are shown in the box.
